# First trimester PAPP-A serum levels and long-term metabolic outcome of mothers and their offspring

**DOI:** 10.1038/s41598-020-61830-5

**Published:** 2020-03-20

**Authors:** Arrigo Fruscalzo, Adriana Cividino, Emma Rossetti, Alessia Maurigh, Ambrogio P. Londero, Lorenza Driul

**Affiliations:** 1 Frauenklinik, Christophorus-Kliniken, Coesfeld, 48653 Germany; 2 Clinic of Obstetrics and Gynecology, University of Münster, Münster, 48149 Germany; 3 Clinic of Obstetrics and Gynecology, Ostalb-Klinikum Aalen, Aalen, 73430 Germany; 4 Clinic of Obstetrics and Gynecology, DAME, University of Udine, ASUI - Presidio Ospedaliero Universitario “SM della Misericordia”, Udine, 33100 Italy; 5 Azienda Sanitaria Universitaria Integrata di Udine, Udine, 33100 Italy

**Keywords:** Predictive markers, Gestational diabetes, Risk factors

## Abstract

Low maternal serum levels of pregnancy associated plasma protein A (PAPP-A) are known to be associated with the development of pregnancy-related complications like small for gestational age infants, intrauterine fetal demise, gestational diabetes and preeclampsia. The study aims to find possible long-term correlations with the development of metabolic and cardiovascular complications in the mothers and their progeny in later life. This is a retrospective cohort study conducted on consecutive unselected women screened for chromosomal anomalies in the first trimester of pregnancy between 2004 and 2010. PAPP-A values as well as clinical data collected at childbirth were considered. A maternal and neonatal follow-up was performed through a telephone interview with the mother during 2015. The body-mass-index and the presence of cardiovascular diseases, dyslipidaemia and diabetes mellitus were evaluated. The analysis included 988 patients. The median time of follow-up was 7 years (IQR 6–9). Lower first trimester maternal blood PAPP-A quartiles were associated with small stature of the offspring (z-score 1st-2nd quartile 0.37 IQR −0.42 and 1.17 vs 3rd-4th quartile 0.67 IQR −0.17 and 1.36, p < 0.05). Furthermore, low first trimester PAPP-A in pregnancy without other gestations following the index one, in Kaplan-Meier analysis was associated to a significant increase of hypoglycemic agents use at 7 and 10 years (respectively 1.12% CI.95 0–2.38% and 5.45% CI.95 0–10.82%) compared to the control group of high first trimester PAPP-A values (0% CI.95 0–0%) (p < 0.05). Low PAPP-A serum levels in the first trimester of pregnancy are associated with short stature in offspring and de-novo development of maternal diabetes mellitus in later life.

## Introduction

PAPP-A, the pregnancy-associated plasma protein A, is produced by the placental syncytiotrophoblast and, interacting with the insulin-like growth factors, plays a critical role in the step of invasion, in the growth of the placenta and the fetus^1–3^. PAPP-A is a metalloproteinase that cleaves three insuline growth factor binding protein (IGFBP):IGFBP-2, -4 and -5; it binds to the surface of the cells and releases bioactive insulin growth factor (IGF) in proximity to their receptor, acting as a growth promoting enzyme^1^. The paracrine/autocrine actions of trophoblastic IGF may be important in the invasion of the endometrium and for the features of both the decidua, and the trophoblast. *In vitro* studies have shown that abundant levels of IGFBP-1 on the decidual side can modulate the secretion of proteases, thus adjusting the invasion.

Several clinical studies indicate that a low concentration of PAPP-A in maternal blood in the first trimester of pregnancy is correlated to placental dysfunction^4–7^. The association between PAPP-A levels in the first trimester of gestation and the development of metabolic complications during pregnancy has already been demonstrated; according to the current literature, however, the possible role of this protein in predicting future maternal complications after pregnancy has not been reviewed yet. The “Barker hypothesis” assigns to the placenta a pivotal role in fetal programming and leads back the development of cardiovascular and metabolic diseases in later life to intrauterine life^8^. In this perspective, we decided to investigate if PAPP-A levels at 11–13 + 6 weeks of gestation can predict the development of metabolic and cardiovascular pathologies in mothers and their offspring in later life.

## Materials and methods

### Study design, setting and participants

In this retrospective study, we reviewed the PAPP-A measurements performed for the first trimester aneuploidy screening and compared them with maternal and children’s clinical data, gathered from obstetrical records and from an interview with the patients years later.

This retrospective cohort consisted of 3263 consecutive pregnant women who underwent the first trimester screening for trisomies in the period between January 2005 and December 2010 at the University of Udine, Italy. Patients who underwent the ultrasound screening, combined with the additional maternal blood examination for commercial markers of chromosomal abnormalities (Pregnancy-Associated Plasma Protein-A -PAPP-A and free beta subunit of Human Chorionic Gonadotropin -free ß-HCG) were considered. Multiple pregnancies were excluded, as were patients with an anomalous fetal cariotype. A telephone survey was conducted after having sent an informed-consent letter to each patient between October 2014 and August 2016. Every participant agreed to answer a post-delivery follow-up interview and to make the retrospective data available for this analysis. PAPP- A serum concentration and clinical data were than retrospectively obtained from the clinical records of the first trimester chromosomal abnormalities screening. The first trimester screening blood samples were collected in the gestational period between 11 weeks and 13 weeks, 6 days of gestation, according to the usual screening protocol. The present research was conducted in accordance with the Helsinki declaration and was approved by the Internal Review Board of the Department of Medical Area (University of Udine).

### Data measurement

The automated platform IMMULITE® (Siemens Healthcare Diagnostics) was used to measure maternal serum PAPP-A and free β-HCG. The IMMULITE® platform determines concentrations using an immunometric assay with solid phase labeled with chemiluminescent enzyme. The resulting measures of PAPP-A and free β-HCG were converted into multiples of the median (MoM) using the software Prisca (Typolog, Germany), after adjusting for factors that could affect the values (maternal weight, tobacco smoking, maternal diabetes mellitus, type of conception, and ethnicity).

### Variables

The following maternal data regarding the index pregnancy were considered: maternal and paternal age, parity (distinguishing nulliparous from women who had already delivered), height, weight and body mass index (BMI) at delivery, macro-region of origin, mode of conception (spontaneous induction, ovulation with or without intrauterine insemination and *in vitro* fertilization), mode of delivery, gestational age at delivery, pregnancy-related hypertensive disorders (PRHDs), gestational and pre-gestational diabetes mellitus (respectively GDM and type I or II DM).

The considered newborn’s data were: sex, birth weight, placental weight, Apgar score at first and fifth minute, intrauterine growth restriction (IUGR), small for gestational age (SGA), large for gestational age (LGA) and malformations.

The indicators chosen for the mothers’ follow up were: BMI (weight - height square ratio), persistence or new onset of diabetes mellitus, persistence or new onset of hypertension or other metabolic diseases after the end of the puerperium (about 6 weeks after childbirth). The indicators chosen for the offspring were the following: BMI (weight - height square ratio), the diagnosis of diabetes mellitus, hypertension and other metabolic diseases. In addition, offspring’s weight, height and BMI z-scores were calculated according to the Centers for Disease Control and Prevention (CDC) growth curves^9^.

The macro-regions of origin were categorized into 6 groups: Italy and Western Europe (which includes the European Union before 2004, Switzerland, Norway and Iceland); Eastern Europe; Asia (which includes Nepal, Bangladesh, Bhutan, Northern Asia, central, Eastern and South Eastern Asia); Arab countries (which includes North Africa, South-western and South Asia, except for Nepal, Bangladesh and Bhutan); Sub-Saharian Africa; and other countries)^10^. Mode of delivery was classified as spontaneous vaginal delivery, operative vaginal delivery and cesarean section. Gestational age at delivery was considered as a continuous or dicotomic value using as significant limit >34 weeks of gestation. Gestational hypertensive diseases include: pre-eclapsia, defined by systolic blood pressure of greater than or equal to 140 mmHg or a diastolic blood pressure of greater than or equal to 90 mmHg in conjunction with proteinuria (defined as the urinary excretion of 0.3 g protein or greater in a 24-hour period, which usually correlates with 30 mg/dl or greater in a random urine determination), gestational hypertension (defined as the same as pre-eclampsia, but without proteinuria), and pre-eclampsia superimposed on chronic hypertension (defined as pre-eclampsia in a patient with hypertension present before the 20th gestational week)^11–14^. Placental index was calculated as the ratio between placental weight and newborn’s weight^10^. The Apgar score was assigned to every newborn at one and five minutes of age, as universally accepted, giving the value 0, 1 or 2 to the following signs and adding them to compute the score, to the following signs: heart rate, respiratory effort, muscle tone, reflex irritability, color^15^. We classified all babies with a weight at birth under the 10th percentile as SGA, while all babies with a birth weight over the 90th percentile were considered LGA^12^. The multiple of the median (MoM) of the neonatal weight was calculated as follows: neonatal weight/50° percentile of the neonatal weight at the same gestational week, adjusted for neonatal sex^16^.

### Statistical analysis

Statistical analysis was performed using R (version 3.5.2). It was considered a significant value of p < 0.05. The normality of the distribution was assessed using the Kolmogorov-Smirnoff test. The following statistic tests were used: in the case of continuous variables Wilcoxon test or t-test; in the case of categorical variables the chi-square test or Fisher’s exact test. A correlation analysis was performed using the Spearman rank correlation test. The population was divided according to the distribution of the adjusted PAPP-A MoM values, comparing the first and second quartile (considered low value) of the distribution with the other two quartiles (considered high value). In addition, a multivariate analysis was performed using logistic regression and considering as dependent variables the significant outcomes and as independent variables the adjusted PAPP-A MoM and the possible confounding factors. Finally, a Kaplan-Meier analysis was carried out and the differences with the Log-Rank test were evaluated.

## Results

We considered 3263 consecutive pregnancies. In total, 2275 cases were excluded for unavailability to the follow-up (missing contact data or consent of the patient to participate to the study). Overall, we included in the current analysis 988 singleton pregnancies. The median time of follow-up was 7 years (IQR 6–9).

### Population’s features

Table 1 shows the characteristics of the population. Mothers were predominantly from Italy and Western Europe, with a mean age at the index pregnancy of 32.68 years (±4.44) and a mean pre-pregnancy BMI of 22.77 kg/m^2^ (±4.06). In 98.38% of the cases delivery occurred after 34 weeks’ gestation. Considering the characteristics of ultrasound screening for aneuploidies performed in the first trimester of pregnancy, the average values were within the normal range. Considering the neonatal characteristics, 49.60% of the neonates were males, 7.19% were SGA and 11.46% were LGA. In addition, 37.45% (370/988) of women had a new pregnancy after the considered index pregnancy.

**Table 1:** (A) Characteristics of the population. (B) Characteristics on ultrasound and biochemical screening of the first trimester of pregnancy. (C) Characteristics of the newborn.

**(A) Population characteristics**	
Maternal age at birth (years)	32.68 (±4.45)
Maternal height (cm)	166.07 (±10.89)
Maternal pre-pregnancy BMI (kg/m²)	22.77 (±4.06)
Nulliparity	42.41% (419/988)
Paternal age at birth (years)	36.02 (±5.53)
Paternal height (cm)	179.11 (±8.25)
Maternal smoking	
Unknown	0.20% (2/988)
No	96.66% (955/988)
Yes	3.14% (31/988)
Family macro area of origin	
Italy and Western Europe	92.3% (911/987)
Eastern Europe	4.26% (42/987)
Sub-Saharan Africa	1.62% (16/987)
Arabian countries	1.11% (11/987)
Asia and other	0.71% (7/987)
Pre-pregnancy diabetes mellitus	1.11% (11/988)
Gestational diabetes	3.24% (32/988)
PRHDs <34 weeks’ gestation	0.61% (6/988)
Preterm birth <34 weeks’ gestation	1.62% (16/988)
Mode of delivery	
Vaginal spontaneous birth	64.37% (636/988)
Vaginal operative birth	9.21% (91/988)
Caesarean section	26.42% (261/988)
**(B) First trimester screening**	
CRL (mm)	58.73 (±6.99)
NT (mm)	1.48 (±0.33)
NT (MoM)	0.97 (±0.19)
PAPP-A (corrected MoM)	1.02 (±0.59)
Free ß-HCG (corrected MoM)	1.21 (±0.88)
**(C) Neonatal characteristics**	
Male neonatal sex	49.6% (490/988)
SGA (<10th percentile)	7.19% (71/988)
LGA (>90th percentile)	11.64% (115/988)
Neonatal weight (grams)	3355.41 (±526.29)
Neonatal weight (MoM)	1.02 (±0.12)
Neonatal length (cm)	50.09 (±2.79)
Neonatal length (MoM)	1.01 (±0.04)

Table 2 displays the maternal and offspring features at follow up. Excluding those ones suffering from pre-gestational diabetes, at the time of follow-up 4.61% of the mothers (45/977) were taking anti-hypertensive, hypoglycemic, or lipid-lowering drugs. In particular, 1.72% (17/988) of all the women were taking hypoglycemic drugs or insulin at the time of follow-up; excluding cases affected by pre-gestational diabetes, the 0.77% (7/977) of women had begun taking these drugs only years after delivery.

**Table 2:** Maternal and offspring features at follow up.

**Offspring characteristics**	
Offspring age (months)	84.9 (72.07–111.02)
Offspring height (cm)	125 (118–135)
Offspring height (z-score)	0.52 (−0.28–1.28)
Offspring weight (Kg)	25 (21–30)
Offspring weight (z-score)	0.27 (−0.37–0.85)
Offspring BMI (Kg/m²)	15.87 (14.58–17.58)
Offspring BMI (z-score)	0.01 (−0.81–0.85)
**Maternal characteristics**	
Use of anti-hypertensive drugs	3.54% (35/988)
Use of lipid-lowering drugs	0.51% (5/988)
Use of oral hypoglycemic agents/insulin	1.72% (17/988)
Use of oral hypoglycemic agents/insulin (*)	0.72% (7/977)
Use of anti-hypertensive, lipid-lowering, or hypoglycemic agents (*)	4.61% (45/977)
Use of oral hypoglycemic agents/insulin (**)	0.98% (6/611)
Use of anti-hypertensive, lipid-lowering, or hypoglycemic agents (**)	5.56% (34/611)
Use of levothyroxine	7.49% (74/988)

(*) Mothers with pre-gestational diabetes are excluded.

(**) Mothers with pre-gestational diabetes are excluded, as well mother with a new pregnancy following the index pregnancy.

Table 3 shows the differences in the characteristics of the population between the low value of PAPP-A (first and second quartile of the distribution) and the other two quartiles. The neonatal length value was not significantly different between low and high PAPP-A values (p = 0.070). In addition, no significant differences were found in maternal characteristics (such as pre-pregnancy BMI or tobacco smoke habits).

**Table 3:** Characteristics of the population, of the first trimester ultrasound and of the newborn, divided by the distribution of PAPP-A corrected MoMs (1st and 2nd quartile vs 3th-4th quartile).

	PAPP-A MoM 1st-2nd quartiles (494)	PAPP-A MoM 3th-4th quartiles (494)	p
Maternal age at birth (years)	32.54 (±4.44)	32.83 (±4.46)	0.310
Maternal height (cm)	166.23 (±14.33)	165.91 (±5.63)	0.646
Maternal pre-pregnancy BMI (kg/m²)	22.93 (±4.13)	22.62 (±3.99)	0.230
Nulliparity	40.49% (200/494)	44.33% (219/494)	0.220
Paternal age at birth (years)	35.75 (±5.73)	36.30 (±5.31)	0.120
Paternal height (cm)	178.78 (±9.45)	179.47 (±6.69)	0.320
Maternal smoking			
Unknown	0.20% (1/494)	0.20% (1/494)	1.000
No	97.37% (481/494)	95.95% (474/494)	0.220
Yes	2.43% (12/494)	3.85% (19/494)	0.200
Family macro area of origin			
Italy and Western Europe	92.51% (457/494)	92.09% (454/493)	0.800
Eastern Europe	4.05% (20/494)	4.46% (22/493)	0.750
Sub-Saharan Africa	2.02% (10/494)	1.22% (6/493)	0.320
Arabian countries	0.81% (4/494)	1.42% (7/493)	0.360
Asia and other	0.61% (3/494)	0.81% (4/493)	0.700
Pre-pregnancy diabetes mellitus	1.82% (9/494)	0.40% (2/494)	0.060
Gestational diabetes	5.26% (26/494)	5.47% (27/494)	0.890
PRHDs <34 weeks’ gestation	1.01% (5/494)	0.20% (1/494)	0.220
Preterm birth <34 weeks’ gestation	2.02% (10/494)	1.21% (6/494)	0.310
Mode of delivery			
Vaginal spontaneous birth	60.73% (300/494)	68.02% (336/494)	<0.05
Vaginal operative birth	9.72% (48/494)	8.70% (43/494)	0.580
Caesarean section	29.55% (146/494)	23.28% (115/494)	<0.05
CRL (mm)	58.19 (±6.88)	59.28 (±7.07)	<0.05
NT (mm)	1.44 (±0.30)	1.52 (±0.35)	<0.05
NT (MoM)	0.95 (±0.18)	0.99 (±0.20)	<0.05
Free ß-HCG (corrected MoM)	1.13 (±0.88)	1.28 (±0.88)	<0.05
Male neonatal sex	53.04% (262/494)	46.15% (228/494)	<0.05
SGA (<10th percentile)	8.70% (43/494)	5.67% (28/494)	0.070
LGA (>90th percentile)	11.94% (59/494)	11.34% (56/494)	0.770
Neonatal weight (grams)	3308.27 (±559.39)	3402.54 (±486.99)	<0.05
Neonatal weight (MoM)	1.01 (±0.13)	1.02 (±0.12)	0.120
Neonatal length (cm)	49.88 (±2.99)	50.29 (±2.56)	<0.05
Neonatal length (MoM)	1.00 (±0.04)	1.01 (±0.04)	0.070

### Maternal long-term outcome

Among patients without pre-gestational diabetes, in the group with low PAPP-A the use of hypoglycemic agents had a higher prevalence 1.24% (6/485) than the group with high PAPP-A 0.20% (1/492) (p = 0.068). To reduce possible biases correlated to pregnancies following the considered index gestation, the patients without subsequent pregnancies up to the follow up time, were stratified: the use of hypoglycemic agents was significantly higher (2.01%) in the low PAPP-A group than in the high PAPP-A group (0%) (p < 0.05). Considering the subgroup without new pregnancies after the index pregnancy, the composite outcome of use of anti-hypertensive, lipid-lowering, or hypoglycemic agents was also more prevalent in low PAPP-A than in high PAPP-A group (7.38% vs 3.83% p = 0.060).

**Figure 1:**
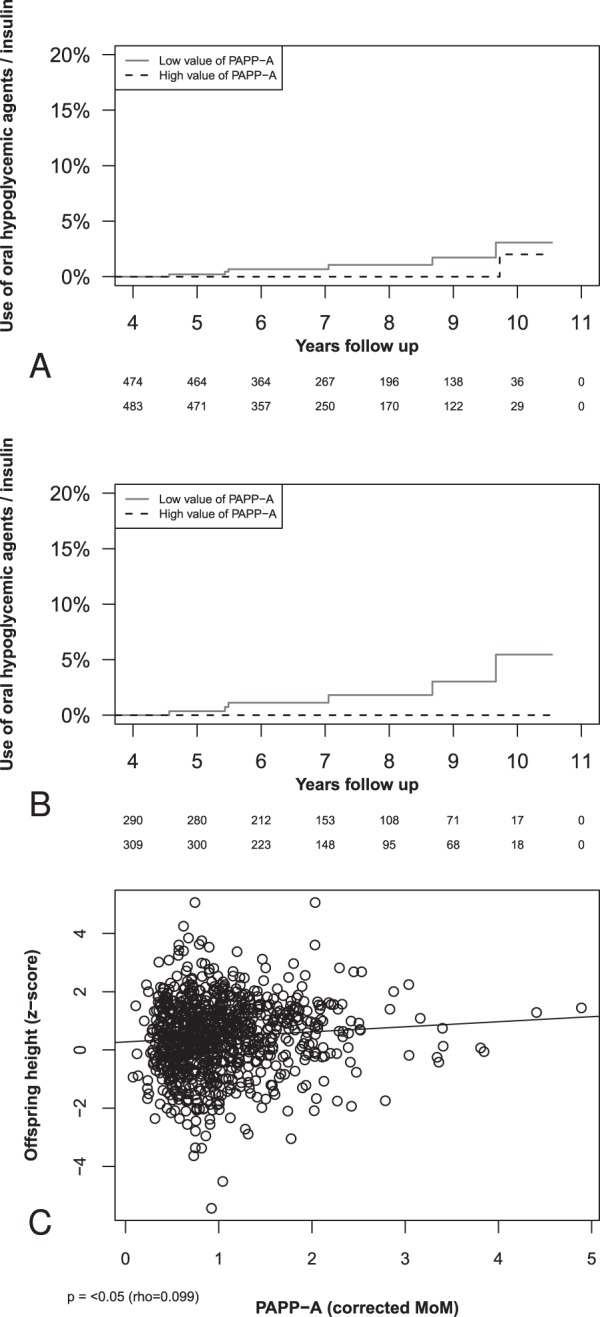
(Panel A) Cumulative events of hypoglycemic agents/insulin use in the years after birth according to the PAPP-A value in the first trimester of the index pregnancy. Log-rank test p = 0.080. It should be noted that cases of pre-gestational diabetes mellitus were excluded prior to analysis. (Panel B) Cumulative events of hypoglycemic agents/insulin use in the years after birth, in the subgroup without pregnancies following the index pregnancy, according to the PAPP-A value in the first trimester of the index pregnancy. Log-rank test p < 0.05. It should be noted that cases of pre-gestational diabetes mellitus were excluded prior to analysis. (Panel C) Correlation between Z scores of offspring height and maternal blood value of PAPP-A corrected MoM in the first trimester of pregnancy.

In Fig. 1A, we highlight the cumulative events based on the time elapsed since birth and it can be seen how a low expression of PAPP-A in the first trimester of pregnancy is associated with an increased use of hypoglycemic agents in the population without pre-gestational diabetes (p = 0.080). Here we also see how, 7 and 10 years after the childbirth, the prevalence of use of these drugs in the group with low PAPP-A was respectively 0.67% (CI.95 0–1.43%) and 3.07% (CI.95 0–6.12%), while in the group of patients with a PAPP-A in the two higher quartiles the prevalence was respectively 0% (CI.95 0–0%) and 2% (CI.95 0–5.8%). Figure 1B shows the data of pregnancy without other gestations following the index one: all the women using hypoglycemic agents belonged to the group with low first trimester PAPP-A values and the difference was statistically significant (p < 0.05). In addition, the prevalence of hypoglycemic agents use 7 years after the childbirth was 1.12% (CI.95 0–2.38%) versus 0% (CI.95 0–0%) in the high first trimester PAPP-A values. Meanwhile, the prevalence of hypoglycemic agents use 10 years after the childbirth was 5.45% (CI.95 0–10.82%) versus 0% (CI.95 0–0%) in the high first trimester PAPP-A values. Considering the multivariate logistic regression, the increasing value of first trimester PAPP-A corrected MoM was significantly protective against the use of hypoglycemic agents even after correction for pre-pregnancy BMI at index pregnancy, maternal age at the index pregnancy, and gestational diabetes at index pregnancy (OR 0.027, CI.95 0.001–0.693, p < 0.05).

### Offspring height

Table 4 shows the differences with regards to the characteristics of the population at the time of follow-up. The group with low expression of PAPP-A presents a significantly lower offspring height z-score (0.37, −0.42–1.17) than the group with high expression of PAPP-A (0.67, −0.17–1.36) (p < 0.05). Figure 1C shows the serum level of PAPP-A in the first trimester of pregnancy is directly correlated with the offspring height z-score at the time of follow-up (rho = 0.099, p < 0.05). Considering the logistic regression analysis, an increasing value of PAPP-A corrected MoM (OR 1.614, CI.95 1.168–2.231, p < 0.05) is significantly associated to an offspring height z-score higher than the median of the distribution, even after correction in the multivariate logistic regression for newborn length MoM, offspring sex, as well as maternal and paternal height (OR 1.654 CI.95 1.184–2.31; p < 0.05).

**Table 4:** Characteristics at follow-up divided for the distribution of PAPP-A corrected MoMs (1st-2nd quartile of the distribution vs 3rd-4th quartile of the distribution).

	PAPP-A MoM 1st-2nd quartiles (494)	PAPP-A MoM 3th-4th quartiles (494)	p
**Offspring characteristics**			
Offspring height (z-score)	0.37 (−0.42–1.17)	0.67 (−0.17–1.36)	<0.05
Offspring weight (z-score)	0.26 (−0.36–0.76)	0.27 (−0.40–0.94)	0.781
Offspring BMI (z-score)	0.03 (−0.75–0.84)	−0.03 (−0.92–0.85)	0.305
**Maternal characteristics**			
Use of anti-hypertensive drugs	3.44% (17/494)	3.64% (18/494)	0.863
Use of lipid-lowering drugs	0.61% (3/494)	0.40% (2/494)	0.654
Use of oral hypoglycemic agents/insulin	3.04% (15/494)	0.40% (2/494)	<0.05
Use of oral hypoglycemic agents/insulin (*)	1.24% (6/485)	0.20% (1/492)	0.068
Use of anti-hypertensive, lipid-lowering, or hypoglycemic agents (*)	4.95% (24/485)	4.27% (21/492)	0.612
Use of oral hypoglycemic agents/insulin (**)	2.01% (6/298)	0.00% (0/313)	<0.05
Use of anti-hypertensive, lipid-lowering, or hypoglycemic agents (**)	7.38% (22/298)	3.83% (12/313)	0.060
Use of levothyroxine	6.28% (31/494)	8.70% (43/494)	0.147

(*) Mothers with pre-gestational diabetes are excluded.

(**) Mothers with pre-gestational diabetes are excluded, as well mother with a new pregnancy following the index pregnancy.

## Discussion

Based on our findings, low maternal serum PAPP-A levels in the first trimester of pregnancy are related to long term outcomes: a small stature of the offspring and a higher prevalence of maternal metabolic diseases among women, measured in terms of hypoglycemic agents intake, after a median of 7 years follow-up.

The most interesting data concern the growth of the offspring and the metabolic diseases of the mother during follow-up. A correlation between the values of PAPP-A in the first trimester of pregnancy and the children’s height has not yet been described in the current literature. This study, to our knowledge, is the first to report such an association. What is already well established is that low values of PAPP-A are correlated with an increased incidence of SGA infants, as we also report^17–21^. Studies have already shown that levels of PAPP-A are related to fetal growth: they are significantly lower in SGA infants compared with appropriate for gestatinal age (AGA) infants, and they are an independent factor in predicting placental weight and birthweight^22,23^. Conversely, a high PAPP-A appears to be associated with a statistically significantly lower prevalence of SGA and with an increased risk of LGAs^24,25^.

The linkage that we demonstrated with height in childhood is consistent with some studies on pregnancy-associated plasma protein A2 (PAPP-A2), which is an enzyme that shares nearly 40% amino acid homology with PAPP-A and acts similarly as an IGFBP protease. Experiments on k/o mice found that PAPP-A2 deficiency compromises fetal growth and skeletal phenotypes and PAPP-A2 human mutations were proven to cause short stature due to low IGF-I availability^26^. Our findings support the insight that PAPP-A has a decisive role in the modulation of bone remodeling^27^. The explanation of our result is still unclear, but could lie in PAPP-A protease activity: this enzyme, degrading inhibitors IGFBP-4 and -5, is able to increase the local bioavailability of the insulin-like growth factors, which are mitogenic and antiapoptotic endocrine factors that play a crucial part in the growth of fetal cells and in placental function^1,7^. According to our results, though, PAPP-A levels not only relate to placental function and intrauterine growth, but also to post-natal development. This correlation between PAPP-A and children’s further growth is yet unexplored; the interpretation could involve fetal programming, through different mechanisms: fetal under-nutrition, glucocorticoid exposure, or genetic and epigenetic links^8,28^. Children could also inherit from their mothers a constitutional low genetic expression of PAPP-A, which is known to be influenced by maternal characteristics, including weight and racial origin^29^. This latter hypothesis could be further investigated measuring PAPP-A serum levels in children and comparing them to maternal values.

The other interesting and newfound aspect of this study is the relationship existing between the low levels of PAPP-A in the first trimester of pregnancy and the development of metabolic diseases such diabetes mellitus in the mother after many years. Considering their pharmacological history, which was investigated through the questionnaire, women with an expression of PAPP-A in the first and second quartile of the distribution show higher rates of disease compared to the population of the third and fourth quartiles, where the concentration of PAPP-A is greater. In particular, this correlation acquires more significance if we consider only the patients who did not have subsequent pregnancies up to the follow up time.

The relationship between the concentrations of PAPP-A and the presence of metabolic disorder during pregnancy it has long been known. The associations between PAPP-A levels and preeclampsia or other PRHDs during pregnancy have been diffusely described: the levels of PAPP-A are lower in pregnancies complicated by PRHD compared to the levels in pregnancies with normal blood pressure, as our data confirmed, hence PAPP-A has been proposed as an early marker for the screening of preeclampsia in combination with other serum proteins and maternal demographics^30–33^.

The relationship with metabolism has been studied especially for Gestational Diabetes Mellitus (GDM). Several studies found that, in the first trimester of pregnancy, the values of PAPP-A levels were lower in patients who developed GDM, compared to patients with normoglycemic pregnant, suggesting a possible role of this biochemical marker in GDM screening in early pregnancy^34,35^. From these data it can be deduced that low PAPP-A concentrations could be an indicator of a pathophysiological process that will lead to GDM and type 2 DM years after pregnancy, but which is already in place months before the disease becomes striking. This hypothesis is supported by Pellitero *et al*., who have explored the influence of glycemic control in the regulation of PAPP-A^36^, looking into the correlation between PAPP-A, inflammatory cytokines and glycemic control in diabetic non-pregnant patients. PAPP-A in fact, as previously described, is not only secreted by the placenta during pregnancy, but also by many other fetal and adult tissues. As an example, consider that in non-pregnant and healthy individuals, the concentration is around 3–5 mIU/L, while at the tenth week of gestation it touches 1000 mIU/L. Returning to the study conducted by Pellitero *et al*., the value of PAPP-A was still lower in diabetic patients compared to the non-diabetic control group, and there was an inversely proportional relationship between the concentrations of PAPP-A and glycated hemoglobin HbA1c.

### Limits of the study

The predictive value of PAPP-A concerning the future development of metabolic complications in the years after the index pregnancy could be explained by the fact that pregnancy may be the revealing event of an early subclinical altered metabolic state, that continues to develop in the subsequent years. This perspective points out pregnancy as a favoured moment in women’s life to implement primary prevention.

Our findings could be widely generalized, as the considered sample is broadly representative, being composed by 929 unselected consecutive pregnancies. Nevertheless, in order to increase statistical sensitivity, further investigation, involving a larger sample, should be conducted in order to verify and further advance our results. The telephone follow up, as depends on the subjective report of the mother about their clinical condition and their children’s health, could have introduced memory and information bias in the study, that we tried to manage asking specifically about any kind of drug therapy instead of about a diagnosis.

Co-morbidities were revealed through the drug history, within the questionnaire, but any disease in remission, for which the patient was not taking any medication, was harder to identify and this could have had an effect on the outcome sought. To minimize these effects, we decided to submit the questionnaires to patients by telephone so that the interviewer was able to ensure the understanding of the question, to receive quickly the required information, and to capture directly any additional information on health status. We obtained follow-up data only from a part of the original cohort (988/3263), but we estimated that the selection bias effect lost to follow up is weak, because the population of participants and non participants were similar.

## Conclusions

Our data show that low PAPP-A serum levels in the first trimester of pregnancy are associated with short stature in offspring and de-novo development of maternal diabetes mellitus in later life. Even if allocating a role of PAPP-A in the screening of future metabolic disease among pregnant women is still undoubtedly premature, we believe that our research may contribute to a better understanding of metabolic disorders. These results should be taken into account by obstetric care professionals in order to inform patients about possible further complications, making it possible to enact prevention measures, hence improving woman’s health.

### Condensation

The low PAPP-A serum levels in the first trimester of pregnancy is associated with short stature in children and later development of diabetes mellitus in the mothers.

## Data Availability

The data that support the findings of this study are available, but restrictions apply to the availability of these data, which was used under license for the current study, and so are not publicly available. Data are however available from the authors upon reasonable request and with permission of the Internal Review Board.
